# The association between abused adults and substance abuse in Taiwan, 2000–2015

**DOI:** 10.1186/s12888-023-04608-z

**Published:** 2023-02-23

**Authors:** Chi-Hsiang Chung, Iau-Jin Lin, Yao-Ching Huang, Chien-An Sun, Wu-Chien Chien, Nian-Sheng Tzeng

**Affiliations:** 1 School of Public Health, National Defense Medical Center, 11490 Taipei, Taiwan; 2 Department of Medical Research, Tri-Service General Hospital, 11490 Taipei, Taiwan; 3 Taiwanese Injury Prevention and Safety Promotion Association (TIPSPA), 11490 Taipei, Taiwan; 4 Department of Chemical Engineering and Biotechnology, National Taipei University of Technology (Taipei Tech), 10608 Taipei, Taiwan; 5 Department of Public Health, College of Medicine, Fu-Jen Catholic University, 242062 New Taipei City, Taiwan; 6 Big Data Center, College of Medicine, Fu-Jen Catholic University, 242062 New Taipei City, Taiwan; 7 Graduate Institute of Life Sciences, National Defense Medical Center, 11490 Taipei, Taiwan; 8 Student Counseling Center, National Defense Medical Center, 11490 Taipei, Taiwan; 9 Department of Psychiatry, Tri-Service General Hospital, 11490 Taipei, Taiwan

**Keywords:** Adult maltreatment, Female, Substance abuse, Violent injury

## Abstract

**Objective:**

To investigate whether adults suffering from violence were at risk of substance abuse and provides insight into the relationship between male and female abusers and substance abuse from 2000 to 2015 in Taiwan.

**Methods:**

This study used data on outpatient, emergency, and inpatient visits for 2 million people enrolled in universal health insurance from 2000 to 2015. ICD-9 diagnosis codes 995.8 (abused adult) and E960–E969 (homicide and injury purposely inflicted by other persons) were defined in this case study, analyzing first-time violence in adults aged 18–64 (study group). Non-abused patients (control group) were matched in a 1:4 ratio, and the paired variables were gender, age (± 1 year), pre-exposure Charlson Comorbidity Index, and year of medical treatment. SAS 9.4 and Cox regression were used for data analysis.

**Results:**

A total of 8,726 people suffered violence (control group: 34,904 people) over 15 years. The prevalence of substance abuse among victims of violence was 78.3/10^4^, 61.9/10^4^, and 51.5/10^4^ for tobacco use disorder, alcoholism, and alcohol abuse, respectively. The risk (adults, overall) of drug abuse, drug dependence, and alcoholism after exposure to violence (average 9 years) was 7.47, 7.15, and 6.86 times (*p* < 0.01), respectively, compared with those without violence. The risk (adults, males) of drug abuse, drug dependence, and alcohol abuse after exposure to violence (average 9 years) was 6.85, 6.27, and 6.07 times, respectively, higher than those without violence (*p* < 0.01). Risks of drug dependence, alcohol abuse and alcoholism (adults, females) after exposure to violence (average 9 years) were 14.92, 12.26, and 11.55 times, respectively, higher than non-abused ones (*p* < 0.01).

**Conclusion:**

The risks of substance abuse, after adult violence, are higher than in those who have not suffered violent injuries.

## Introduction

Abuse is a common problem in societies around the world and can be defined as acts that intentionally harm or damage an individual physically, psychologically, or socially [1]. In the case of adult abuse, an individual does or says something to hurt, disturb, or frighten anyone over the age of 18 [2]. Abuse can happen anywhere, and anyone can commit it [2]. Some abused adults find it harder to get help and maybe are more vulnerable to harm and exploitation because of conditions such as disability, poor mental health, and temporary or long-term illness. Other adults at risk of abuse may be frail older adults or caregivers, such as a partner, relative, friend, and so on [3]. There are many types of abuse, such as Physical Abuse (intentionally causing physical pain or harm), Psychological Abuse (deliberately causing emotional distress through verbal assault, threats, intimidation, humiliation or other means), Sexual Abuse (unwelcome sexual activity), Neglect (lack of necessary help to maintain physical and mental health), and so on [4]. A 2005 study in the United States (US) found that male violence against female partners was associated with cohabitation and witnessing parental domestic violence; female violence against male partners was associated with male unemployment, witnessing parental domestic violence, and alcohol abuse; in multivariate analysis results, alcoholism was the only indicator associated with intimate partner violence [5].

Abuse has severe, lasting negative emotional, mental and physical effects on the victim [6]. These include increased risk of substance abuse, including tobacco use disorder, alcoholism, alcohol abuse, drug dependence, and drug abuse [5, 6]. Substance abuse is caused by prolonged exposure to substances and subsequent mental and physical dependence, which can lead to social and occupational barriers and negative health effects [7]. Substance abuse is a major public health problem affecting all segments of society. Individual, household, community, and overall government spending are affected by legal and illicit drug use [8]. Recent reports estimate that in the US, the approximate annual cost of illicit drugs, excessive alcohol consumption, and tobacco use is $193 billion, $223 billion, and $193 billion, respectively [9]. These costs include lost wages and productivity, criminal activities, and medical expenses [10, 11]. Alcohol, tobacco, marijuana, and, increasingly, prescription drugs are the most commonly abused substances across age groups; however, use patterns and marked effects of these and other drugs vary by life stage [12]. In terms of health outcomes, all age groups are at risk for overdose, unintentional injury, and suicide attempts [8].

A better understanding of the physical and mental health effects on adults who experience violence and substance abuse can aid public health efforts. Currently, there are limited longitudinal observational studies on the relationship between adult abuse and substance abuse. Therefore, we hypothesized that adult female patients exposed to violence are at the greatest risk of drug dependence. We used the Ministry of Health and Welfare’s National Health Insurance Research Database (NHIRD) to track whether adults suffering from violence were at risk of substance abuse and provides insight into the relationship between male and female abusers and substance abuse from 2000 to 2015 in Taiwan through long-term follow-up.

## Method

### Data sources

Taiwan officially implemented National Health Insurance (NHI) on March 1, 1995. According to the statistics of the Gender Equality Committee of the Executive Yuan, the actual coverage rate of NHI has increased from 99.29% to 1998 to 99.93% in 2020. This study utilized the NHIRD to provide a representative NHI 2000 parent cohort with 2 million coverage sample files (Longitudinal Health Insurance Research Database, LHID2000) as the study data source, and the follow-up period ranged from January 1, 2000, to December 31, 2015, that is, six years of outpatient and inpatient data. In this study, the data from 2000 were used for data cleaning, and non-new cases were excluded. The adults who suffered violent abuse in this study were 18 to 64 years old, defined according to the International Classification of Diseases, Ninth Revision, Clinical Revision (ICD-9-CM) diagnosis codes 995.8 and E960–E969. Thus, the abused adult group did not have prior experience with substance abuse.

The Charlson Comorbidity Index (CCI) used in this study was a modified version [13]. Types of injury according to ICD9 diagnosis codes 800–989, 995.80–995.85 (Adult Abuse). This study used Taiwan’s NHIRD. The analyzed data do not contain personally identifiable information. The Ethics Review Board of the National Defense Medical Center Tri-Services General Hospital (TSGHIRB number: C202105014) approved this study, waiving individual written informed consent. The source of funding for the project is the scientific research project of the General Hospital of the National Defense Medical College: TSGH-B-112,020/ TSGH-E-112,259.

### Study design

We performed a prospective matched cohort design. From January 1, 2000, to December 31, 2015, ICD-9-CM diagnosis code 995.80 was used for claims of adult abuse. Victims over the age of 18 were included in the adult abuse cohort (n = 8,726, experimental group). In addition, 34,904 participants’ (control group) comparison cohorts, without any experience of adult abuse, matched (1:4) for gender, age, CCI, and index date. The sampling source of the control group was the non-abused patients, and the control group was established according to the matching ratio of 1:4. Time of first exposure to violence (month and year, the same year and month of treatment in the control group), CCI. Among them, adjustments were made to the CCI prior to the acts of violence. In order to be able to find enough control people, the CCIs above 10 points were classified into the same level according to the actual situation of the data, and the rest were matched according to the original CCI scores. After the two groups of data were paired according to the abovementioned matching conditions, the same pairing numbers were given to those with the same matching conditions for the statistical analysis of the paired data. Substance abuse includes tobacco use disorder, alcoholism, alcohol abuse, drug dependence, and drug abuse. Victims with experience of adult or substance abuse before the index date or 2000 were excluded.

Covariates in this study included gender, age, geographic region of residence (northern, central, southern, or eastern Taiwan), urbanization level (grades 1–4), hospital level (medical center and regional and district hospital), and insurance fee category​​​ (New Taiwan Dollars [NTD]; < 16,500, 16,500–30,299, ≥ 30,300).

The level of urbanization is based on population density (person/km^2^), the percentage of the population with a college education or above (%), the percentage of the population over the age of 65 (%), the percentage of the population classified as agricultural workers (%), and the number of physicians per 100,000 people in the county [14].

Comorbidities include attention deficit hyperactivity disorder, intellectual disability, autism/pervasive developmental disorder, conduct disorder/oppositional defiant disorder, other developmental disorders, childhood mood disorders, Tourette’s syndrome/tic disorder, and enuresis/stool incontinence.

This study applied the CCI score with 17 relevant comorbidity categories (based on ICD-9-CM diagnosis codes) [15]. CCI scores ranged from 0 to 37, indicating no comorbidities caused by serious health problems.

We defined these five categories of substance abuse through ICD-9 as follows:

First, tobacco use disorder (ICD-9: 305.1) in which one is addicted to tobacco. With this disorder, one has trouble stopping using tobacco [16]. Tobacco contains the drug nicotine, which is addictive because it quickly boosts one’s mood. Subsequently, one wants to use it more, which makes it hard to stop, even though one is aware of the risks [17].

Second, alcoholism (ICD-9: 303) is broadly any act of drinking alcohol that results in significant mental or physical health problems [18]. There is disagreement on the definition of the word alcoholism; it is not a recognized diagnostic entity [18]. Predominant diagnostic classifications are alcohol use disorder (DSM-5) or alcohol dependence (ICD-11); these are defined in their respective sources [19].

Third, alcohol abuse (ICD-9: 305.0) encompasses a spectrum of unhealthy alcohol-drinking behaviors ranging from binge drinking to alcohol dependence and, in extreme cases, resulting in health problems for individuals and large-scale social problems such as alcohol-related crimes [20].

Fourth, drug dependence (ICD-9: 304) is a chronic, progressive disease characterized by significant impairment that is directly associated with persistent and excessive use of a psychoactive substance [21].

Fifth, drug abuse (ICD-9: 305.2-305.9) refers to the use of illegal drugs or the use of prescription or over-the-counter drugs for purposes other than those for which they are meant to be used, or in excessive amounts [22].

**Fig. 1:**
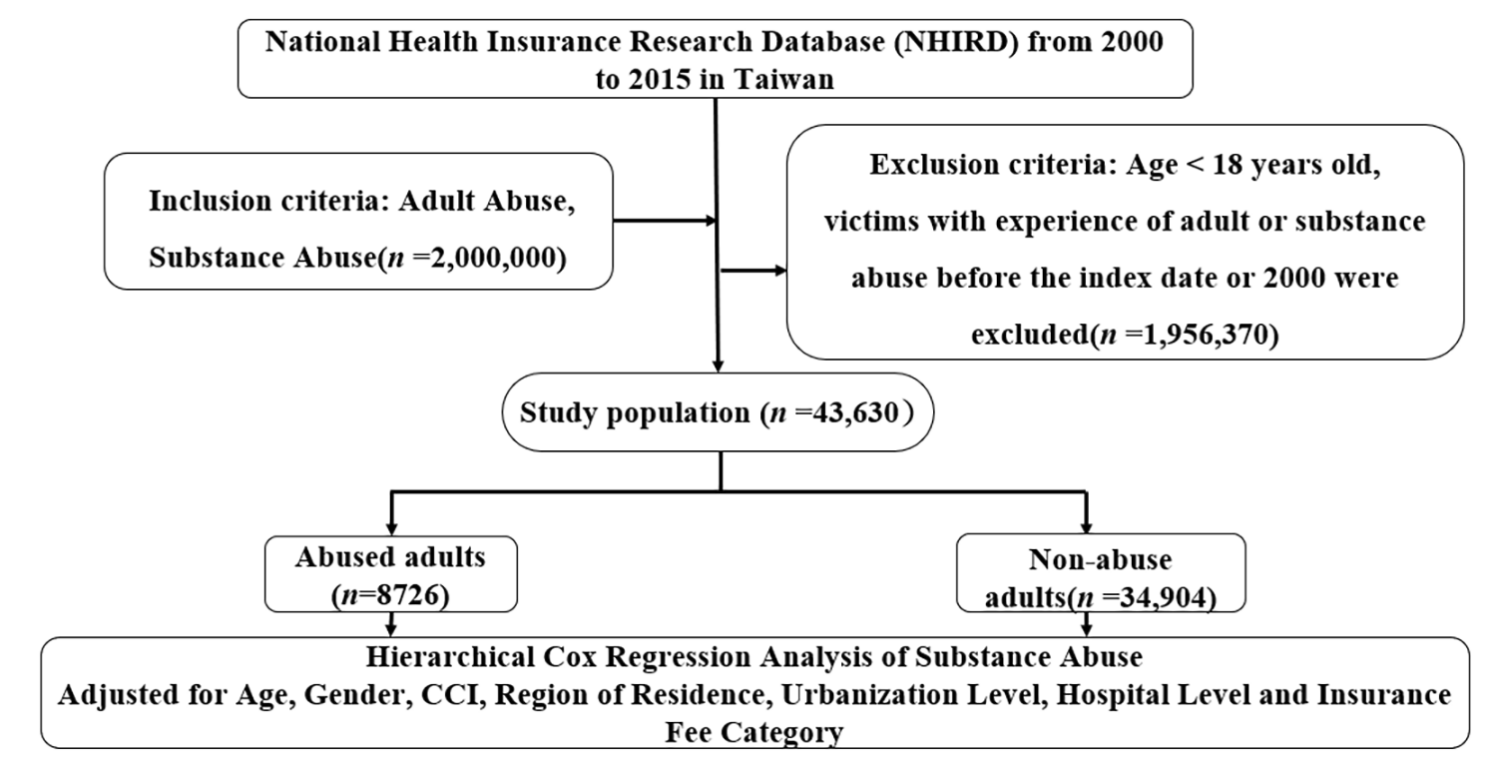
The flowchart of the study sample selection

The study follow-up period was from January 1, 2000, until the onset of mental illness and substance abuse, withdrawal from the NHI program, or until the end of 2015. Figure 1 shows the study design flow chart for this study.

### Statistical analysis

This study was analyzed using SAS 9.4 for Windows (SAS Institute, Cary, NC, USA) provided by the Academia Sinica Branch of the Data Welfare Center of the Ministry of Health and Welfare, using chi-square test, hierarchical Cox regression, paired Subsequently, the descriptive data of the two groups were tested using the GEE method.

## Results

### Characteristics of study

Table 1 presents the differences in patient characteristics between the two groups in this study. A total of 43,630 adults included a experimental group of 8,726 abused adults and a matched control group of 34,904 non-abuse adults. The average age of abused adults was 36.9 ± 12.0 years, including 5853 male patients (67.1%) and 2873 female patients (32.9%). The differences between the two groups in this study were in the following variables: insurance premiums, CCI, urbanization level, and care level.

**Table 1:** Basic demographic data of adult victims and control groups seeking medical treatment

Variables		Experimental Group (*n* = 8726)			Control Group (*n* = 34,904)		*p-value*
		*n*	*%*		*n*	*%*	
Gender	Male	5853	**67.1**		23,412	67.1	> 0.999
	Female	2873	32.9		11,492	32.9	
Age	Mean (SD)	36.9	(12.4)		36.9	(12.2)	0.927
Level of Urbanization	High	1758	**20.1**		10,157	29.1	< 0.001**
	Moderate	2513	28.8		11,255	32.2	
	Low	4455	51.1		13,492	38.7	
Area of Insurance	North	3260	37.4		17,849	51.2	< 0.001**
	Central	2408	27.6		6238	17.9	
	South	2771	31.7		9738	27.9	
	Eastern	287	3.3		1079	3.1	
Amount of Insurance	< 16,500 (NTD)	2870	32.9		5894	16.9	< 0.001**
	16,500–30,299 (NTD)	4470	51.2		17,672	50.6	
	≧ 30,300 (NTD)	1386	15.9		11,338	32.5	
Health Insurance category	Public Insurance	233	2.7		1997	5.7	< 0.001**
	Labor Insurance	4542	52.1		23,870	68.4	
	Other Insurance	3951	45.2		9037	26.0	

Note: by *GEE*, correlation matrix: unstructured, *: *p* < 0.05, **: *p* < 0.01

### Risk of mental illness and poor prognosis according to adult maltreatment exposure

Adult victims of violence have significantly higher risks of substance abuse than non-abused patients. Among them, the three adverse outcomes with the greatest risk difference were drug abuse (HR = 7.47), drug dependence (HR = 7.15), and alcohol abuse (HR = 6.86) (Table 2).

**Table 2:** Comparison of the risk of substance abuse between victims of violence and non-abused patients (Adults, Overall)

Poor prognosis	Abused patients (*n* = 8,726)	Non-abused patients (*n* = 34,904)	*HR (95% CI)*	*p-value*	
	incidence (1/10^4^)	incidence (1/10^4^)			
Tobacco use disorder	78.3	44.5	1.75 (1.57–1.94)	< 0.0001**	
Alcoholism	61.9	11.5	5.62 (4.82–6.55)	< 0.0001**	
Alcohol abuse	51.5	7.7	6.86 (5.74–8.22)	< 0.0001**	
Drug dependence	25.0	3.6	7.15 (5.51–9.28)	< 0.0001**	
Drug abuse	16.8	2.3	7.47 (5.43–10.27)	< 0.0001**	

Note: For *stratified Cox regression* analysis, corrected CCI, insurance amount, insurance status, gender, age, and CCI before pairing have been corrected at the time of pairing. The reference group comprised the non-abused patients. *: *p* < 0.05, **: *p* < 0.01

When divided by gender, adult male victims of violence have significantly higher risks of substance abuse than non-abused patients. Among them, the three adverse outcomes with the largest risk difference were drug abuse (HR = 6.85), drug dependence (HR = 6.27), and alcohol abuse (HR = 6.07) (Table 3).

**Table 3:** Comparison of the risk of substance abuse between victims of violence and those without violence (Adults, Males)

Poor prognosis	Abused male patients (*n* = 5,853)				Non-abused male patients (*n* = 23,412)				*HR* *(95% CI)*	*p-value*
	*n*	%	Total person-years	incidence (1/10^4^)	*n*	%	Total person-years	incidence (1/10^4^)		
Tobacco use disorder	457	7.8	47,696	95.8	1135	4.8	192,627	58.9	1.61 (1.44–1.81)	< 0.0001**
Alcoholism	343	5.9	47,244	72.6	297	1.3	193,965	15.3	5.01 (4.25–5.91)	< 0.0001**
Alcohol abuse	273	4.7	47,509	57.5	191	0.8	194,348	9.8	6.07 (4.99–7.39)	< 0.0001**
Drug dependence	137	2.3	48,209	28.4	92	0.4	194,472	4.7	6.27 (4.74–8.31)	< 0.0001**
Drug abuse	83	1.4	48,206	17.2	52	0.2	194,544	2.7	6.85 (4.77–9.85)	< 0.0001**

Note: For *stratified Cox regression* analysis, corrected CCI, insurance amount, insurance status, gender, age, and CCI before pairing have been corrected at the time of pairing. The reference group was the non-abused patients. *: *p* < 0.05, **: *p* < 0.01

Adult female victims of violence have significantly higher risks of substance abuse than non-abused women. Among them, the three adverse outcomes with the largest risk difference were drug dependence (HR = 14.92), alcohol abuse (HR = 12.26), and alcoholism (HR = 11.55) (Table 4).

**Table 4:** Comparison of the risk of substance abuse between victims of violence and those without violence (Adults, Females)

Poor prognosis	Abused female patients (*n* = 2,873)				Non-abused female patients (*n* = 11,492)				*HR (95% CI)*	*p-value*
	n	%	Total person-years	incidence (1/10^4^)	n	%	Total person-years	incidence (1/10^4^)		
Tobacco use disorder	83	2.9	21,259	39.0	107	0.9	86,582	12.4	3.25 (2.39–4.41)	< 0.0001**
Alcoholism	80	2.8	21,120	37.9	27	0.2	86,646	3.1	11.55 (7.40–18.03)	< 0.0001**
Alcohol abuse	81	2.8	21,207	38.2	26	0.2	86,676	3.0	12.26 (7.74–19.42)	< 0.0001**
Drug dependence	37	1.3	21,303	17.4	10	0.1	86,704	1.2	14.92 (7.15–31.11)	< 0.0001**
Drug abuse	34	1.2	21,384	15.9	12	0.1	86,719	1.4	9.82 (5.03–19.17)	< 0.0001**

Note: For *stratified Cox regression* analysis, corrected CCI, insurance amount, insurance status, gender, age, and CCI before pairing have been corrected at the time of pairing. The reference group were the non-abused patients. *: *p* < 0.05, **: *p* < 0.01

## Discussion

The results of this study show that the risk of substance abuse in adults who have been subjected to violence is significantly higher than in those who have not been subjected to violence. Adult female patients exposed to violence are at the greatest risk of drug dependence. The top three risks of substance abuse (adults, overall) were drug abuse, drug dependence, and alcoholism. The top three risks of substance abuse (adults, males) were drug abuse, drug dependence, and alcohol abuse. The top three risks of substance abuse (adults, females) were drug dependence, alcohol abuse, and alcoholism. Previous research found that 19.5 million women (15.4%; aged 18 and above) had used illicit* drugs in the past year [23]. Women who experience domestic violence are at an increased risk of drug dependence, which is consistent with our study [24]. Women often use different substances than men, such as using small amounts of certain drugs for a shorter period before becoming addicted [25]. Women may respond differently to substances; they may have more drug cravings and are more likely to relapse after treatment [26]. Sex hormones can make women more sensitive to the effects of certain drugs than men [27]. Women who use the drug may also experience more physical effects on the heart and blood vessels [28]. Brain-chemistry changes may be different in women who use drugs than in men; women are more likely to go to the emergency room or die from an overdose of certain substances or other effects [29]. Divorce, loss of child custody, or the death of a partner or child can trigger substance use or other mental health disorders in women [30]. Women who use certain substances may be more prone to panic attacks, anxiety, and depression [31].

From a gender perspective, some patterns of different phenomena can be identified in drug dependence between genders [32]. Among consumers, women were the most likely to take sedatives, tranquilizers, and other drugs (reflecting data from the drug-treated population), while men were associated more with marijuana and heroin use [33]. A World Health Organization report also details the impact of intimate violence on women’s physical, mental, sexual and reproductive health: it does highlight that women who have been sexually or physically abused by their partners report higher levels of health problems and that they are likely to seek medication for consolation [34]. Substance abuse plays a major role in making addicts more vulnerable, drugs and other substances used by victims represent a risk factor that increases the risk of being assaulted, and women affected by drugs may be more vulnerable [35]. After experiencing sexual assault, women may look for a strategy to deal with physical, mental, and emotional problems: The negative effects of sexual assault drive individuals to seek behaviors that can quickly reduce negative emotions, Substance use or abuse may represent coping after exposure to violence strategies to reduce the amount of physical and subjective disgust [36, 37]. In summary, our study found that the risk of substance abuse in adults who have been subjected to violence being significantly higher than those who have not been subjected to violence.

The study has several limitations that warrant consideration. First, similar to the previous study using the NHIRD on psychiatric disorders [38], we were unable to evaluate the genetic factor, psychosocial factor, environmental factor, severity, or psychological assessments in the patients with psychiatric disorders, as the data were not recorded in the database. Second, the subjects of this study are adults who have been exposed to violence and seek medical care. Therefore, only those who have experienced more severe violence will be observed by the investigator, and the incidence may be underestimated. Third, this study only observed cases of patients seeking medical treatment after experiencing violence, which may lead to underreporting the actual number of cases of injury due to violence, especially domestic violence cases, and people who have suffered psychological abuse without obvious trauma, or other unidentified reasons and violent cases in which patients do not dare to seek medical treatment. Finally, due to significant differences in basic attributes of such cases, individuals are more likely to be at risk of drug abuse, drug dependence, and alcohol dependence rather than experience violent risk factor effects.

## Conclusion

The results of this follow-up study support a strong association between the risk of substance abuse in adults who have been subjected to violence being significantly higher than those who have not been subjected to violence. Therefore, in addition to striving to avoid violent incidents, medical and social welfare personnel should pay attention to the mental problems of violent adults, as well as the risk of substance abuse (especially drug abuse, drug dependence, and alcoholism).

Future studies should investigate whether there have been any changes in the poor prognosis, including drug abuse, drug dependence, and alcoholism, over the observation period from 2016 to 2022.

## Data Availability

The datasets generated during and analyzed during the current study are not publicly available due to legal restrictions imposed by the government of Taiwan concerning the“Personal Information Protection Act” but are available from the corresponding author on reasonable request.
